# Eyebrow tattoo-associated sarcoidosis: A case report

**DOI:** 10.3389/fmed.2022.1009135

**Published:** 2022-11-18

**Authors:** Shu Nie, Ke Li, Chuang Gao, Na Yin, Zile Chen, Zhouwei Wu

**Affiliations:** Department of Dermatology, Shanghai General Hospital, School of Medicine, Shanghai Jiao Tong University, Shanghai, China

**Keywords:** sarcoidosis, permanent makeup, tattoo, granulomas, eyebrow

## Abstract

Cutaneous sarcoidosis can manifest after doing a permanent makeup (PMU), such as tattooed eyebrows. A 41-year-old Chinese woman, with a tattoo in the eyebrows, developed yellow–brown plaques in her eyebrows for several months. A dermatopathological examination revealed non-caseating granulomas consistent with cutaneous sarcoidosis. For months, topical corticosteroids were applied, which showed little effect. Furthermore, a physical evaluation of the patient revealed no apparent involvement of other body organs except bilateral hilar lymphadenopathy with few diffuse reticulonodular opacities. On the basis of fully informed consent, the patient agreed to a 6-month initial follow-up to avoid unnecessary PMU.

## Introduction

Sarcoidosis is an inflammatory granulomatous disease characterized by non-caseating epithelioid granulomas in multiple organs, for which the etiology and pathophysiology are unidentified. In roughly a quarter of cases, cutaneous sarcoidosis is the initial manifestation of the disease (1, 2). Over the past decade, studies have documented on reporting granuloma nodosum on tattoos or permanent makeup (PMU) (3–5). The popularity of tattoos and PMU is on the rise. Though tattoos are generally considered harmless, nonetheless, adverse reactions, namely, allergic reactions, infections, or (systemic) autoimmune diseases can occur. Furthermore, PMU can be a sarcoidosis trigger, and the prevalence of sarcoidosis may increase as its popularity grows. Granuloma and nodular tattoo reactions can be the initial and occasionally the only skin manifestations of systemic sarcoidosis (6). Specifically, we reported a 41-year-old Chinese woman who developed yellow–brown plaques in her eyebrows months after receiving a tattoo in the same location.

## Case presentation

A 41-year-old Chinese woman presented to our clinics with a slowly enlarged rash on the bilateral eyebrows, which had developed for several months without itching or tenderness. The patient exhibited no other symptoms, such as coughing.

**FIGURE 1:**
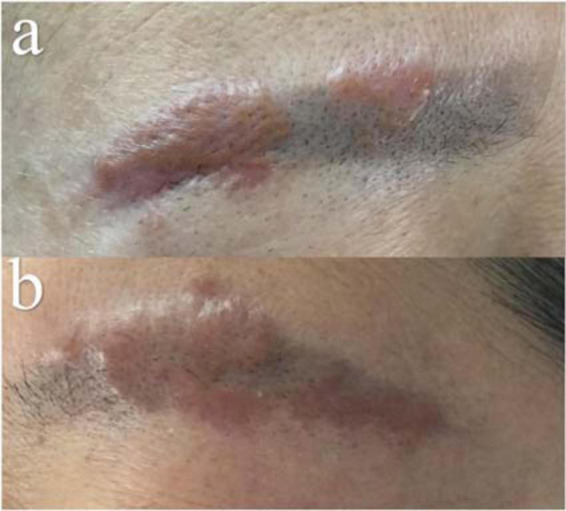
Clinical manifestation. Reddish and yellow–brown plaque within the areas of previous black–brown eyebrow tattoos [Right **(a)** and Left **(b)**].

On physical examination, extensive black–brown tattoos on both eyebrows were observed. There were grouped reddish and yellow–brown infiltrative papules, some of which coalesced into plaques scattered over the tattoo (Figures 1a,b). There were no scales or ulcers found.

The patient disclosed that she had tattooed her eyebrows 3 months before the rash appeared. She was treated with topical clobetasol propionate cream for over 2 months, nevertheless, there was no notable improvement. She denied any history of trauma, sojourn, or contact with animals. Prior to her dermatology clinic visit, she had no other sarcoidosis-related signs or symptoms. No sarcoidosis-related family history was reported.

**FIGURE 2:**
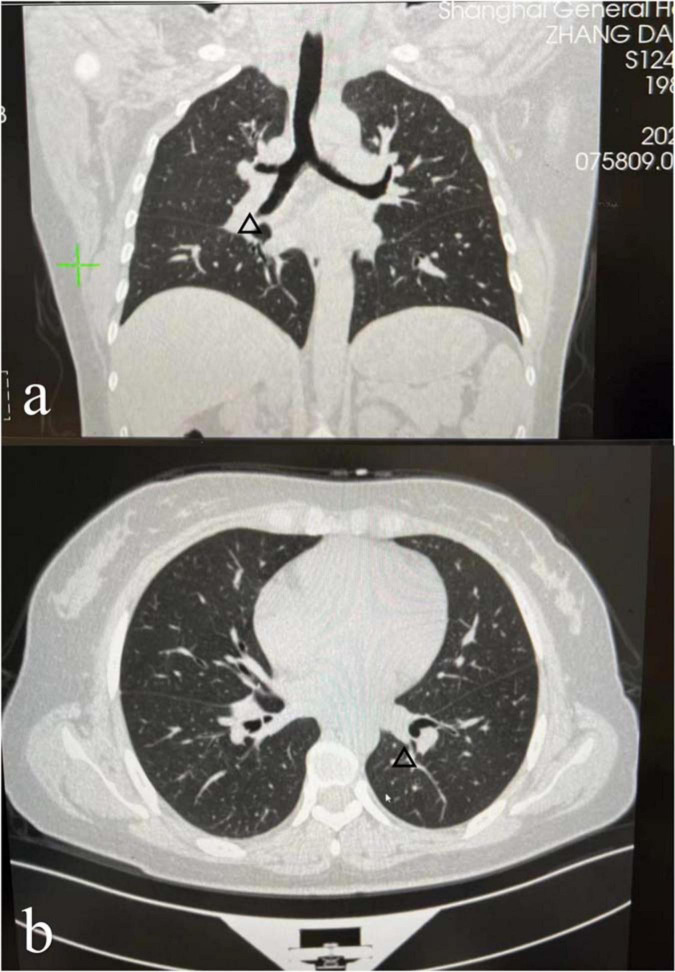
Enhanced computed tomography (CT) of the chest with contrast demonstrated a few diffuse reticulonodular opacities in the bilateral lower lobe, bilateral hilar lymph nodes enlargement [the arrows in panels **(a,b)**], and in the posterior segment of the right upper lobe are some small ground glass nodules.

The results of laboratory tests such as serum angiotensin-converting enzyme (ACE) levels and T-spot were within normal limits (Table 1). Enhanced computed tomography (CT) of the chest with contrast demonstrated a few diffuse reticulonodular opacities in the bilateral lower lobe, bilateral hilar lymph nodes enlargement, and in the posterior segment of the right upper lobe are some small ground glass nodules (Figures 2a,b). The patient declined additional invasive diagnostic procedures, such as bronchoscopy/BAL or EBUS for lymphadenopathy.

**TABLE 1:** The details about routine investigations.

Item	Results	Reference ranges
CBC	7.11 × 10^9^/L	3.69–9.16 × 10^9^/L
ESR	9mm/H	0–20 mm/H
CRP	1.2mg/L	0–10 mg/L
ACE	40.07U/L	12–68 U/L
T-SPOT	Neg	Neg

**FIGURE 3:**
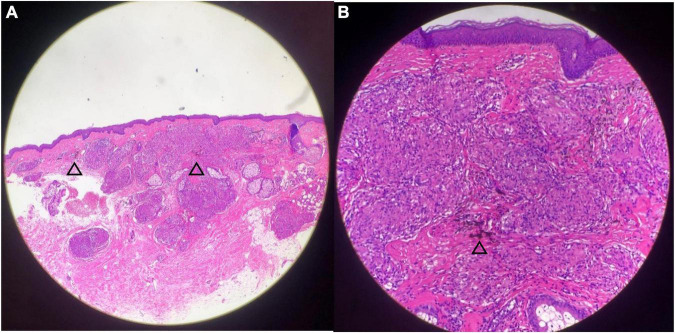
Histopathological examination demonstrated non-caseating granulomas with pigment granules and mild lymphocytic infiltration. Black tattoo pigment is seen throughout the granuloma [the arrows in panels **(A,B)**] [Hematoxylin–Eosin, original magnification ×4 **(A)**, ×20 **(B)**].

A biopsy of yellow–brown plaque within the eyebrow tattoo indicated non-caseating granulomas throughout the dermis consistent with a diagnosis of cutaneous sarcoidosis (Figures 3A,B). Grocott–Gomori methylamine silver stain acid-fast bacilli were negative. Throughout the granuloma, black tattoo pigment was discovered (the arrows in Figures 3A,B).

Tattoo-associated sarcoidosis was diagnosed based on the clinical manifestation of yellow–brown plaque and histopathological findings of non-caseating granulomas at the tattoo site. Lung involvement is considered stage I (1). Since the patient’s symptoms did not enhance following topical steroids, we have decided to follow up with patients every 6 months to avoid unnecessary tattoos (1).

## Discussion

Approximately, 10–20% of the global population is tattooed (7). Notwithstanding, due to the influence of traditional culture in China, tattoos are predominantly employed for PMU, especially eyebrow tattoos. Thus, the negative effects of eyebrow makeup are increasing in China. Uncertain is the precise mechanism of sarcoidosis in eyebrow makeup. It has been reported that chronic and minimal ink exposure may stimulate the granulomatous response and may eventually lead to the development of sarcoidosis in genetically susceptible individuals (8). Our patient showed normal ACE levels, a known sarcoidosis marker (1). However, an elevated ACE level is not diagnostic and a normal level does not rule out sarcoidosis. Surgery, corticosteroids (systemic, topical, intralesional), minocycline, and other treatments are used to treat sarcoidosis, but results are inconsistent (1, 9). The clinical manifestations, natural history, and prognosis of sarcoidosis vary widely, with spontaneous regression, or ebb and flow of disease fluctuations, or in response to therapy. Approximately two-thirds of patients experience spontaneous remission within 12–36 months, whereas 10–30% of patients have chronic or progressive disease (10, 11). Severe extra-pulmonary involvement (mainly heart, nervous system, and liver) has been reported in approximately 4–7% of patients (1). When there is pulmonary fibrosis, it can be life-threatening, with pulmonary hypertension, or cardiac involvement (12, 13). Given that all patients with cutaneous sarcoidosis are likely to develop systemic involvement, it is necessary to assess the risks of morphologically related systemic involvement of cutaneous sarcoidosis (14). Macules and papules with the least correlation with systemic disorders, and typically have a favorable prognosis. Annular lesions or plaques have poor prognoses and a higher risk of systemic involvement. Lupus pernio had a more chronic course than plaque sarcoidosis and correlated significantly with upper respiratory tract and bone involvement. In addition, Heerfordt’s syndrome always has a poor prognosis (14, 15). After 2 months of topical corticosteroid treatment, our patient did not improve significantly. Considering that approximately two-thirds of patients undergo spontaneous remission within 12–36 months after communicating with the patient, we agreed to follow up on the changes of the condition in the next 6 months with skin and chest CT examination, and avoiding unnecessary PMU was advised.

Our patient had an eyebrow tattoo with the purpose of seeking beauty and yet got the opposite result, and the treatment result was not satisfactory. We are aware that PMU may contain more complementary colors and shape alterations than conventional tattoos. Theoretically, repeated tattooing and reintroduction of foreign material into the skin may activate the immune system (16). Clinicians have begun to recognize and predict potential adverse effects in patients with PMU. Detailed clinical information is essential to deliver appropriate treatment to this patient population (17). These patients might represent subclinical cases or cases predisposed to sarcoidosis later in life and consequently, must be monitored. Limited information is available on PMU complications. Existing research consists primarily of case reports and case series with few original studies, and treatment guidelines are still lacking. Furthermore, there is an urgent need for market oversight and restrictions on tattoo ink, as well as the monitoring of tattoo complications.

## Data availability statement

The original contributions presented in this study are included in the article/supplementary material, further inquiries can be directed to the corresponding author.

## Ethics statement

Ethical review and approval was not required for the study on human participants in accordance with the local legislation and institutional requirements. The patients/participants provided their written informed consent to participate in this study. Written informed consent was obtained from the individual for the publication of any potentially identifiable images or data included in this article.

## Author contributions

SN: data collection, formal analysis, and writing—original draft. KL, CG, NY, ZC: writing—review and editing. ZW: conceptualization, resources, and writing—review and editing. All authors contributed to the article and approved the submitted version.
